# The C-Terminal Putative Nuclear Localization Sequence of BReast cancer Metastasis Suppressor 1, BRMS1, Is Necessary for Metastasis Suppression

**DOI:** 10.1371/journal.pone.0055966

**Published:** 2013-02-04

**Authors:** Douglas R. Hurst, Yi Xie, John W. Thomas, Jianzhong Liu, Mick D. Edmonds, Mark D. Stewart, Danny R. Welch

**Affiliations:** 1 Department of Pathology, University of Alabama at Birmingham, Birmingham, Alabama, United States of America; 2 Comprehensive Cancer Center, University of Alabama at Birmingham, Birmingham, Alabama, United States of America; 3 Department of Cancer Biology, Kansas University Medical Center, Kansas City, Kansas, United States of America; University of South Alabama, United States of America

## Abstract

Breast cancer metastasis suppressor 1 (BRMS1) is a predominantly nuclear protein that suppresses metastasis in multiple human and murine carcinoma cell lines. BRMS1 interacts with several nuclear proteins including SIN3:HDAC chromatin remodeling complexes that are involved in repressing transcription. However, recent reports suggest BRMS1 may function in the cytoplasm. BRMS1 has two predicted nuclear localization sequences (NLS) that are located near the C-terminus (amino acids 198–205 and 238–244, NLS1 and NLS2 respectively). We hypothesized that nuclear localization sequences of BRMS1 were essential for BRMS1 mediated metastasis suppression. Replacement of NLS2 with NLS1 (BRMS1^NLS1,1^), truncation at 238 (BRMS1^ΔNLS2^), or switching the location of NLS1 and NLS2 (BRMS1^NLS2,1^) did not affect nuclear localization; but, replacement of NLS1 with NLS2 (BRMS1^NLS2,2^) or truncation at 197 (BRMS1^ΔNLS^ which removes both NLS) promoted cytoplasmic localization. MDA-MB-231 human metastatic breast cancer cells transduced with BRMS1^NLS1,1^, BRMS1^NLS2,2^ or BRMS1^NLS2,1^ were evaluated for metastasis suppression in an experimental xenograft mouse model. Interestingly, while NLS2 was not necessary for nuclear localization, it was found to be important for metastasis suppression since BRMS1^NLS2,2^ suppressed metastasis by 85%. In contrast, BRMS1^NLS2,1^ and BRMS1^NLS1,1^ did not significantly suppress metastasis. Both BRMS1 and BRMS1^NLS2,2^ co-immunoprecipitated with SIN3A in the nucleus and cytoplasm; however, BRMS1^NLS1,1^ and BRMS1^NLS2,1^ were associated with SIN3A in the nucleus only. Moreover, BRMS1 and BRMS1^NLS2,2^, but not BRMS1^NLS1,1^ and BRMS1^NLS2,1^, down-regulated the pro-metastatic microRNA, miR-10b. Together, these data demonstrate an important role for NLS2 in the cytoplasm that is critical for metastasis suppression and is distinct from nuclear localization.

## Introduction

Molecules regulating gene transcription either directly or indirectly have the potential to dramatically impact the metastatic process. Since the discovery of the metastasis suppressor BRMS1 in 2000 [1], there have been multiple proteome and transcriptome studies demonstrating that BRMS1 alters the expression of both coding and non-coding metastasis associated genes [2]–[5]. The coordinated expression of genetic programs is necessary to enable a cancer cell to complete all the required steps of the metastatic cascade [6]–[9]. Although there is no evidence for BRMS1 functioning as a transcription factor, there have been concrete studies showing association with transcriptional repressive chromatin remodeling complexes (reviewed in [10]). BRMS1 presumably regulates transcription by interaction with SIN3:HDAC chromatin remodeling complexes through the direct interaction with AT rich interacting domain 4A (ARID4A) and suppressor of defective silencing 3 (SUDS3) leading to the suppression of basal transcription [11]–[13]. These findings have been confirmed by protein-protein interaction studies of SIN3 complexes and identification of BRMS1 by mass spectroscopy [14]–[20].

As a transcriptional regulatory molecule, it is not surprising that BRMS1 has been involved with modulation of multiple molecular pathways associated with metastasis. In fact, it has been suggested that BRMS1 robustly blocks the overall process of metastasis through small, albeit significant, inhibition of each step in the metastatic cascade [10]. Although this has complicated the studies regarding molecular mechanisms, BRMS1 has been demonstrated to alter specific cellular pathways associated with metastasis including gap junctional intercellular communication [21]–[23], phosphoinositide signaling [24], [25], nuclear factor kappa B signaling [26]–[29], cell motility and invasion [30]–[32], apoptosis [28], [33], and tumor cell dissemination [33]. Because it interacts with SIN3 complexes, it is presumed that BRMS1 is modulating these pathways through transcriptional regulation of critical genes. However, recent data have emerged identifying BRMS1 in the cytoplasm of cells suggesting functions other than transcriptional regulation [34], [35]. In fact, a recent clinical study of malignant melanoma suggested that localization of BRMS1 in the cytoplasm inhibits tumor progression and nuclear BRMS1 actually promotes melanoma cell invasion [36]. These cytoplasmic functions of BRMS1 are not currently understood.

To begin exploring possible cytoplasmic roles, we generated mutations at the two nuclear localization (NLS) regions. We were surprised to find that, although NLS2 was not important for active transport into the nucleus, it was critical for metastasis suppression. We identified potential cytoplasmic functions of BRMS1 through interaction with SIN3A that correlates with the ability of BRMS1 to suppress metastasis. This study adds to our understanding of the BRMS1 metastasis suppressor protein that will expand our knowledge of metastatic disease.

## Experimental Procedures

### Ethics statement

All animal studies were carried out in strict accordance with the recommendations in the Guide for the Care and Use of Laboratory Animals of the National Institutes of Health. The protocol was approved by the University of Alabama at Birmingham Institutional Animal Care and Use Committee (protocol #071106666). Animals were sacrificed by cervical dislocation following anesthesia (ketamine/xylazine) and all efforts were made to minimize suffering.

### Cell lines and cell culture

The metastatic human breast carcinoma cell line, MDA-MB-231, and those constitutively expressing BRMS1 or BRMS1 mutants was described previously [11]. The monkey kidney cell line, COS7, was used for transient transfections as described previously [37]. Immortalized human breast epithelial cell line MCF10A and a metastatic variant MCF10CAa.1 were described previously [38]. All cells were cultured in a mixture (1∶1, v/v) of Dulbecco's modified minimum essential medium and Ham's F12 medium (DMEM/F12; Invitrogen, Carlsbad, CA) supplemented with 2 mM L-glutamine (Invitrogen), 0.02 mM non-essential amino acids (Mediatech, Herndon, VA), and 5% fetal bovine serum (FBS; Invitrogen). Media for MCF10A cells were additionally supplemented with 10 ng/ml EGF, 500 ng/ml hydrocortisone, 100 ng/ml cholera toxins, and 10 µg/ml insulin (Sigma-Aldrich, St. Louis, MO). Cells were passaged at 80–90% confluence using a 0.125% trypsin and 2 mM EDTA solution (MDA-MB-231 and derivatives) or a 2 mM EDTA solution (COS7) in Ca^2+^/Mg^2+^ free Dulbecco's phosphate buffered saline (CMF-DPBS). Cells were maintained on 100 mm tissue culture dishes (Corning, Corning, NY) at 37°C with 5% CO_2_ in a humidified atmosphere. Neither antibiotics nor antimycotics were used and all cell lines were found to be negative for *Mycoplasma spp*. contamination using a PCR-based method (TaKaRa, Shiga, Japan) or PlasmoTest kit (InvivoGen, San Diego, CA).

### Constructs and transfections

**Figure 1:**
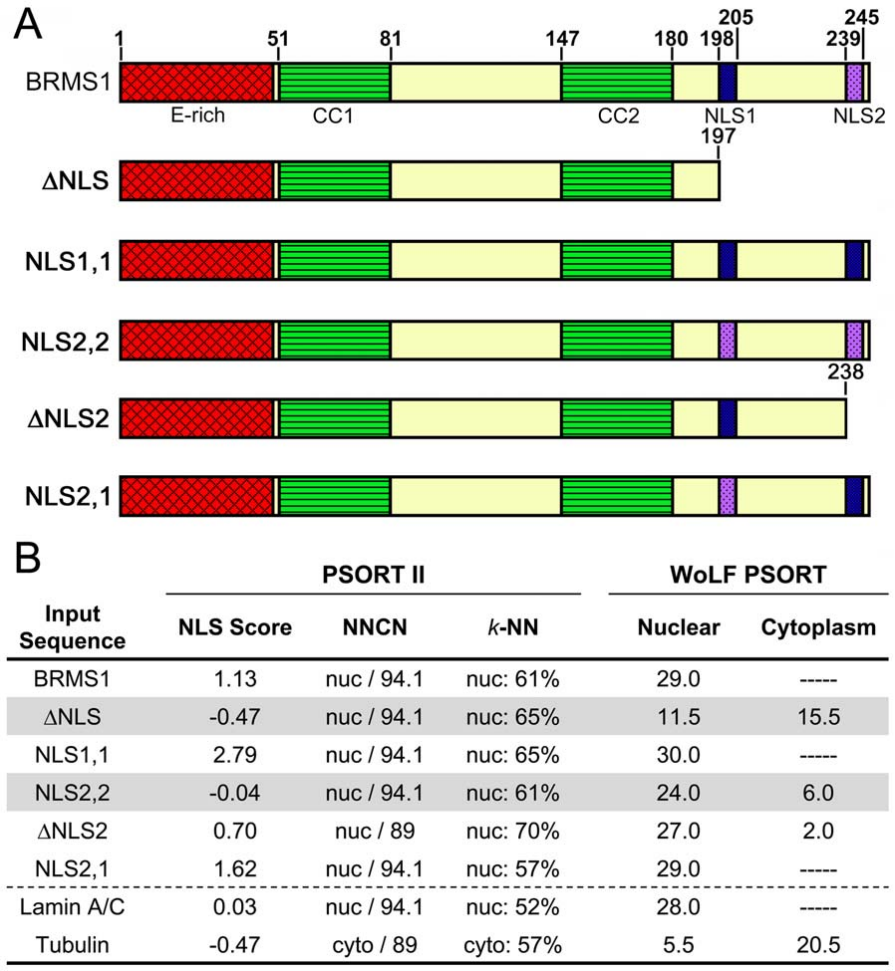
A. Domain organization of BRMS1 and the BRMS1 mutants generated for this study. E-rich is glutamate rich region, CC1 and CC2 are coiled-coil regions, NLS1 and NLS2 are nuclear localization sequences. B. Predicted subcellular localization based on PSORT II and WoLF PSORT (ExPASY proteomics server). The two mutants lacking NLS1 (ΔNLS2 and NLS2,2) have a negative NLS score using PSORT II and high cytoplasmic score using WoLF PSORT. Lamin A/C and tubulin are also listed for proteins representing nuclear and cytoplasmic localization respectively.

GFP-GST fused BRMS1 or mutants were generated by amplifying GST-BRMS1 by PCR from pGEX-2TK/BRMS1 vector. The product was cloned into Gateway system entry vector pENTR/SD/D-TOPO according to the user manual (Invitrogen). Recombination between pENTR/SD/D-TOPO/BRMS1 and destination vector pcDNA-DEST53 was done to generate pcDNA-DEST53/GST-BRMS1 expression vector. BRMS1 mutants were created by QuickChange II site-directed mutagenesis (Stratagene, La Jolla, CA) as described previously [11]. The following GFP-GST fused mutants were generated: truncation of BRMS1 at AA197 (ΔNLS), truncation at AA238 (ΔNLS2), replacement of NLS2 with NLS1 (NLS1,1), replacement of NLS1 with NLS2 (NLS2,2), and switching the location of NLS1 and NLS2 (NLS2,1) (Fig. 1A). All constructs were confirmed by DNA sequencing. COS7 cells were grown on sterilized coverslips without any coating in 12 well-plates. After overnight incubation, transfection was accomplished with lipofectamine 2000 according to the manufacturer's protocol (Invitrogen). The coverslips were removed and fixed with 4% PFA (paraformaldehyde) for 20 min. at room temperature and washed with PBS.

The metastatic human breast carcinoma cell line, MDA-MB-231, was transduced with a HIV type 1-based, lentiviral vector system to constitutively express BRMS1 or BRMS1 mutants as described previously [11]. Lentiviral constructs containing the BRMS1 mutants NLS1,1, NLS2,2, and NLS2,1 without the GFP-GST fusion were used. Three clones of each were selected for experimental metastasis assays.

### Antibodies, co-immunoprecipitation, and immunoblotting

The monoclonal antibody directed against BRMS1 (3a1.21) was described previously [37]. Co-immunoprecipitation and immunoblotting from nuclear and cytoplasmic fractions (NE-PER kit, Thermo Fisher Scientific) using the polyclonal SIN3A antibody (Abcam, Cambridge, MA) was performed as described previously [37], [38]. Anti-lamin A/C polyclonal antibody (Cell Signaling Technology, Danvers, MA) was used for nuclear fraction control and anti-GAPDH (Cell Signaling Technology) as a control for cytoplasmic fractions.

### Fluorescence microscopy

The transfected COS7 cells with GFP-GST-BRMS1 or -mutants were permeabilized with 1% Triton X-100 for 10 min. at room temperature. Blocking was accomplished with 10% BSA in PBS for 30 min. after 3×5 min. washes with PBS. Texas Red conjugated anti-phalloidin antibody (Molecular Probes, Eugene, OR) 1∶2,000 dilution in 2% BSA in PBS was incubated for 1 hr. at room temperature. After 3×5 min. washes with PBS, the coverslips were inverted on DAPI-containing mount media (Vector laboratories, Burlingame, CA), sealed with nail polish, and observed under a Nikon eclipse TE 2000-U fluorescent microscope.

### Proximity ligation assay (PLA)

Interaction of endogenous BRMS1 and SIN3A in MCF10A and MCF10CAa.1 cell lines were analyzed by PLA according to the manufacturer's protocol (Olink Bioscience, Uppsala, Sweden). Briefly, cells were seeded on 8-well chamber slides for 24 hours, fixed with 4% paraformaldehyde, permeabilized with 0.5% triton X-100, and blocked with Duolink blocking solution. The antibodies for SIN3A and BRMS1 were incubated at 1∶100 dilution and the anti-mouse minus and anti-rabbit plus probes were used at 1∶5 dilution. Images were taken as described for fluorescence microscopy.

### Quantitative real-time RT-PCR

miRNA expression was determined as described previously [4], [39].

### Metastasis assays

Experimental metastasis assays (tail vein injection) with athymic mice (10 per group) were performed as previously described [39]. Animals were maintained under the guidelines of the NIH and the University of Alabama at Birmingham Institutional Animal Care and Use Committee. Food and water were provided *ad libitum*.

### Statistical analysis

The number of lung metastases was compared for BRMS1- and BRMS1 mutant-transduced cell lines to the parental MDA-MB-231 line. A Kruskal-Wallis ANOVA of ranks procedure was used with Dunn's post hoc test. All calculations were performed using SigmaStat (SPSS). Statistical significance was defined as a probability p≤0.05.

## Results

### NLS1 is required for nuclear localization

The functional properties of BRMS1 protein are not currently well understood. BRMS1 has a glutamate-rich region, two coiled-coil regions important for protein-protein interactions, and two nuclear localization sequences (NLS) (Fig. 1A). An additional nuclear export sequence was identified within AA74–91 by Rivera *et al*. [34]. In that study, the NLS1 sequence, but not NLS2, was demonstrated to be necessary and sufficient for nuclear localization of BRMS1.

We hypothesized that NLS1 would be required for BRMS1 function as a metastasis suppressor and generated several mutations with alterations of NLS1 and NLS2 (Fig. 1A). The BRMS1 mutant proteins were analyzed by PSORT II and WoLF PSORT (ExPASY proteomics server, http://expasy.org) for cellular localization prediction. The two mutant proteins that are lacking NLS1 (ΔNLS and NLS2,2) had negative NLS scores using PSORT II and had significant cytoplasmic prediction with WoLF PSORT (Fig. 1B). These predictions are consistent with data from Riviera *et al.* [34].

**Figure 2:**
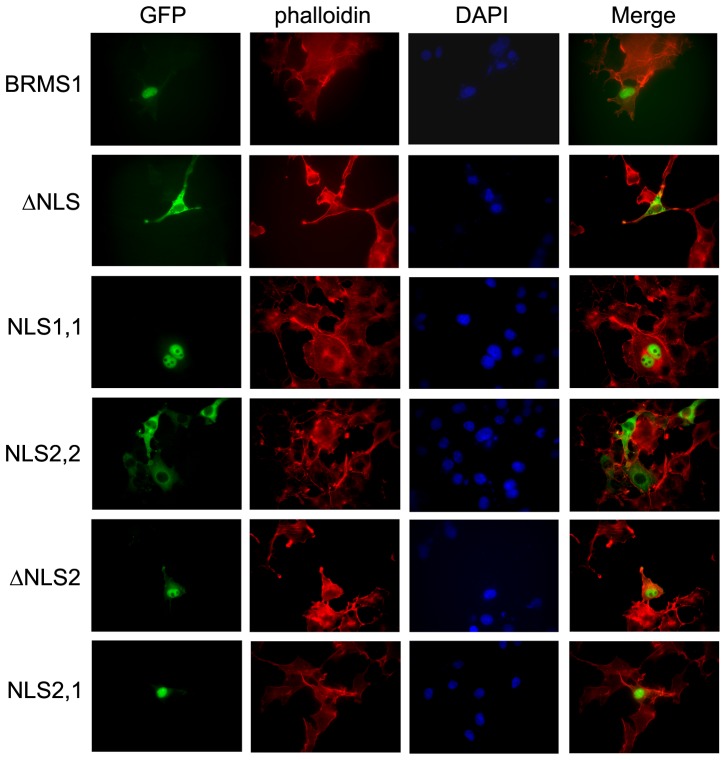
NLS1 is required for nuclear localization. GFP-GST fused constructs listed in Fig. 1 were visualized in COS7 cells. GFP fluorescence from the fused BRMS1 or BRMS1 mutant proteins indicates localization. As predicted, mutants lacking NLS1 were predominantly localized to the cytoplasm. Phalloidin and DAPI were used to visualize the cytoplasm and nucleus respectively.

To validate these predictions, GFP-GST fusion proteins were generated with BRMS1 and the BRMS1 mutants. These chimeric proteins are too large to passively diffuse into the nucleus. GFP fluorescence from transiently transfected COS7 cells was visualized to indicate wild-type BRMS1 and BRMS1 mutant localization (Fig. 2). Phalloidin and DAPI were used for cytoplasmic and nuclear staining respectively. As predicted, mutants lacking NLS1 localized in the cytoplasm demonstrating that NLS1 is both necessary and sufficient for active transport into the nucleus. Nuclear localization of ΔNLS2 additionally shows that NLS1 and NLS2 are not a bipartite nuclear localization signal.

### NLS2 is required for metastasis suppression

**Figure 3:**
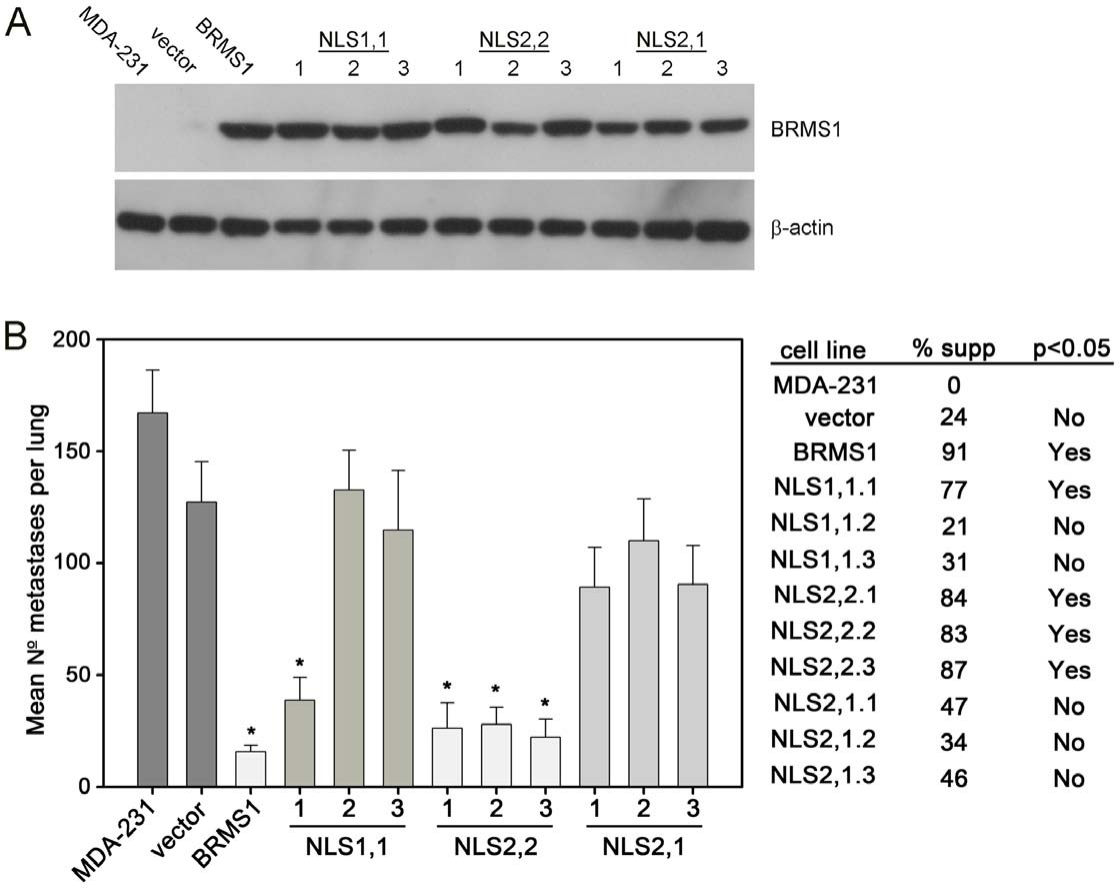
NLS2 is required for metastasis suppression. Three clones each of NLS1,1, NLS2,2, and NLS2,1 were selected for experimental metastasis assays by injection into the lateral tail vein of athymic mice (10 mice per group). A. Western blot of whole cell lysate showing similar levels of expression for BRMS1 and the BRMS1 mutants (β-actin used as loading control). B. The average number of lung metastasis is shown with SEM. The percentage of metastasis suppression is listed on the right. Mutants lacking NLS2 (NLS1,1 and NLS2,1) did not consistently suppress metastasis. * indicates p≤0.05.

**Figure 4:**
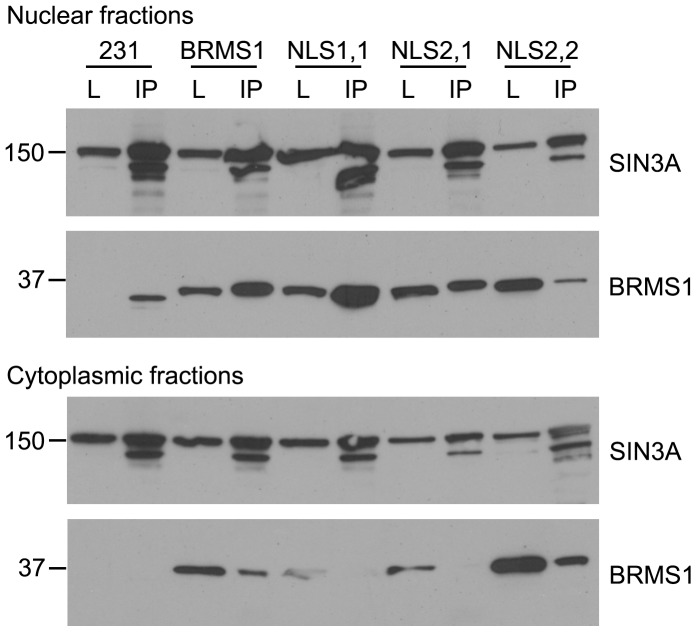
NLS2 is important for cytoplasmic association with SIN3A. Western blot from co-immunoprecipitated samples of SIN3A in nuclear (top panel) and cytoplasmic (bottom panel) fractions are shown. The antibody used to probe the blot is listed on the right and approximate molecular mass on the left. All BRMS1 mutants were detected in the nuclear fractions but only the wild-type and NLS2,2 mutant were precipitated in the cytoplasm. We note that although BRMS1 is usually undetectable by western blot, endogenous BRMS1 could be detected in the nucleus from the co-immunoprecipitation. L is lysate and IP is immunoprecipitation.

The NLS1,1, NLS2,2, and NLS2,1 constructs were chosen to test altered function, specifically metastasis. Since this combination of mutants represented both nuclear and cytoplasmic localization, they also provided the opportunity to explore the impact of subcellular localization on function. We note, however, that GFP-GST chimeras were not utilized in order to minimize any confounding effects these additions would have on metastasis. MDA-MB-231 human metastatic breast carcinoma cells were transduced with lentiviral constructs of NLS1,1, NLS2,2, and NLS2,1 as was previously done with BRMS1. Three cell clones of each transduced mutation were selected for the assays with similar expression levels (Fig. 3A). Because BRMS1 is a relatively small protein (predicted 28.5 kDa, migrates ∼35 kDa with SDS-PAGE) it can still readily passively diffuse into the nucleus. All of the BRMS1 mutant proteins were located in both the nucleus and cytoplasm (Fig. 4 and data not shown).

Three NLS2,2 expressing clones suppressed metastasis at a level comparable to wild-type BRMS1 (Fig. 3B). Neither NLS1,1 nor NLS2,1 significantly suppressed metastasis. This data demonstrates that the NLS2 region at the C-terminus is critical for metastasis suppression and suggests a necessary function distinct from nuclear localization.

### Interactions of SIN3A in the cytoplasm correlates with metastasis suppression

Multiple studies have demonstrated interaction of SIN3A with BRMS1. To begin to understand why NLS2 may be required to suppress metastasis, we immunoprecipitated SIN3A from nuclear lysates and probed for BRMS1 association. All the BRMS1 mutant proteins associated with SIN3A (Fig. 4). Likewise, SIN3A:BRMS1 interactions were present in cytoplasmic fractions for both the wild-type and NLS2,2 mutant but not the NLS1,1 or NLS2,1 mutant (Fig. 4). Thus, NLS2 is required for cytoplasmic association with SIN3A, which also correlates with the ability of BRMS1 to suppress metastasis.

**Figure 5:**
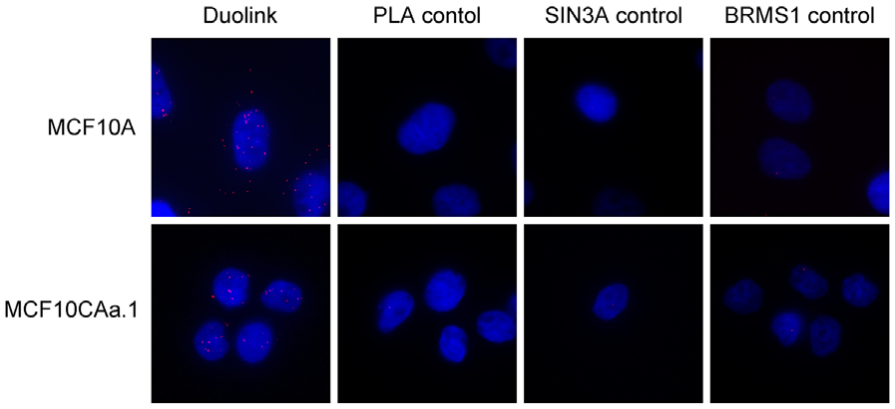
Endogenous interactions of SIN3A and BRMS1 in the cytoplasm. Proximity ligation assays were used to clearly show cytoplasmic interactions in the MCF10A normal “immortalized” breast epithelial cells. The interactions of SIN3A with BRMS1 are predominantly nuclear in the metastatic breast cancer cell line MCF10CAa.1. Each red dot indicates an interaction. Representative images are shown. DAPI is shown in blue. Duolink represents images with both primary antibodies and PLA probes, PLA control is without primary antibodies, SIN3A control is without BRMS1 primary antibody and BRMS1 control is without SIN3A primary antibody.

These interactions were validated by proximity ligation assays using MCF10A and MCF10CAa.1 cell lines (Fig. 5). Endogenous cytoplasmic interactions of SIN3A with BRMS1 are clearly visible in the normal breast epithelial cell line MCF10A; however, the interactions are predominantly nuclear in the metastatic cell line MCF10CAa.1. The cytoplasmic ratio was determined by counting the number of dots outside of the nucleus divided by the total number of dots. MCF10A cells had a cytoplasmic ratio of 59% compared to 22% in the MCF10CAa.1 cells.

### NLS2 is important for miR-10b down regulation

**Figure 6:**
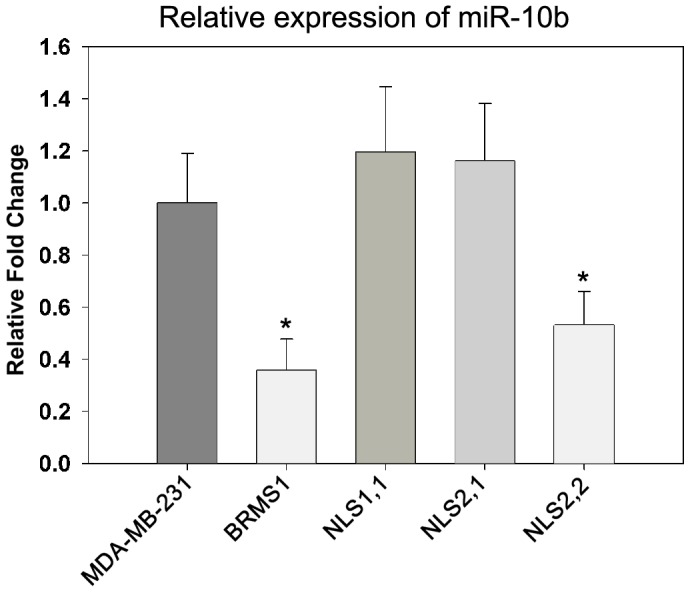
NLS2 is important for down-regulation of miR-10b. Quantitative real-time RT-PCR was used to evaluate the expression levels of miR-10b normalized to the endogenous control RNU6B. Relative expression is shown normalized to the parent MDA-MB-231 cells. Only the wild-type and NLS2,2 mutant decreased the level of miR-10b. * indicates p≤0.05.

To identify whether there were any downstream targets transcriptionally regulated by the BRMS1 mutant proteins; we performed breast cancer disease specific arrays (DSA™, Almac Diagnostics, Durham, NC). Although there were many changes in gene expression between the groups, hierarchical clustering analyses did not reveal correlations to the metastasis data (data not shown) indicating that other modifiers of gene expression may be involved. These arrays include>60,000 transcripts; however, small RNA including miRNA are not included. We therefore proceeded with analyzing selected miRNA associated with metastasis (metastamir) that are known to be regulated by BRMS1 including miR-146a, -146b, and -10b [4], [39]. miR-10b, a pro-metastatic miRNA, was significantly down-regulated by both BRMS1 and the NLS2,2 mutant but not by NLS1,1 or NLS2,1 (Fig. 6).

## Discussion

We hypothesized that nuclear localization of BRMS1 would be required for metastasis suppressive function. Several mutations were generated in order to test this hypothesis; and, surprisingly, we found the second so-called NLS, which does not play a role in active transport into the nucleus, was critical for metastasis suppression. This was contradictory to our original hypothesis but suggested important functional requirements for BRMS1. Somewhat unexpectedly, BRMS1 interacted with SIN3A in the cytoplasm. Concomitantly, the pro-metastatic gene, miR-10b, was down-regulated by both wild-type and the NLS2,2 BRMS1 mutant protein. Taken together, these data demonstrate critical functional requirements of NLS2 in the BRMS1 protein. Moreover, the implication is that BRMS1 protein interactions in the cytoplasm may be, at least partially, responsible for metastasis suppression.

The interpretation regarding cytoplasmic roles for BRMS1 is supported by recent clinical reports [40], [41]. Interestingly, cytoplasmic localization may correlate with good or bad prognosis, depending upon tumor type. Slipicevic et al. present data suggesting that cytoplasmic BRMS1 inhibits melanoma progression and nuclear BRMS1 promotes melanoma invasion [36], [42]. In contrast, Frolova et al. show the opposite trend in breast carcinoma although significance was not reached [40]. A definitive explanation for this discrepancy is not yet proffered; however, we speculate that the size and composition of BRMS1 complexes vary in individual tissues. More comprehensive analyses of complexes will be required in order to fully interpret these data. Corroborating these notions, we recently reported that BRMS1 expression alone was not sufficient to suppress metastasis using mouse models of metastatic mammary carcinoma [43].

It is clear from data published by Rivera *et al*. [34] and in this report that NLS2 is not important for active transport of BRMS1 to the nucleus. Based upon the data reported here, NLS2 (in the correct location) is critical for metastasis suppression and interactions between SIN3A with BRMS1 in the cytoplasm. However, NLS2 is not required for BRMS1:SIN3A associations in the nucleus. It is difficult to understand how these interactions could be differentially occurring based on intracellular location unless one considers the overall composition of these complexes. Specific protein associations with SIN3A may be significantly influenced by tethering proteins. The most logical explanation is that one or more tethering proteins involved in this complex interaction are influencing the BRMS1:SIN3A association. We are currently analyzing SIN3A complex composition in both the nuclear and cytoplasmic fractions to address this hypothesis. Clearly, more structural studies will be required to understand how these proteins are interacting and how these interactions influence function.

One clone expressing the NLS1,1 mutation did not metastasize as well as the MDA-MB-231 parental cells. The simplest explanation relates to heterogeneity within the MDA-MB-231 population (i.e., some cells are metastatic whereas others are not). The poorly metastasizing clone with NLS1,1 was most likely not metastatic to begin with. Although heterogeneity is the likely explanation, we conservatively interpret the findings as NLS1,1 does not consistently suppress metastasis. This is an important consideration and the reason why it is essential to assess multiple cell clones when analyzing tumors, especially for their capacity to metastasize [44]. Similar findings were published when analyzing the metastasis data for cells expressing SUDS3 [45].

Composition of SIN3 complexes dictates function [46], [47]. We have previously shown altered repressive activity with BRMS1 mutants that associate with SIN3 complex components differentially [11]. The regulation of chromatin remodeling by SIN3A complexes has been relatively well-characterized; however, functions in the cytoplasm have, to the best of our knowledge, not been explored. Cytoplasmic localization has been demonstrated using multiple techniques, including immunofluorescence, immunoprecipitation with western blot and mass spectroscopy, and proximity ligation assays (manuscript in preparation). The fact that only wild-type BRMS1 and the NLS2,2 mutant interact with cytoplasmic SIN3A suggests important functions distinct from chromatin condensation and transcriptional repression that are involved in metastasis suppression. In addition to HDAC enzymes being recruited to SIN3, other protein modifying enzymes have been associated [47]. It is conceivable that BRMS1 could modulate the association of protein modifying enzymes to SIN3A in the cytoplasm that affect multiple pathways involved in metastasis. These protein modifying enzymes could significantly alter important post-translational modifications leading to functional changes of key signaling molecules. Additionally, these complexes could be affecting the stability or degradation of small RNA including miRNA. BRMS1 alters the expression of many metastasis-associated miRNA or metastamir [48], [49]. It is interesting that only wild-type BRMS1 and the NLS2,2 mutant had the ability to down-regulate the pro-metastatic miR-10b. Further mechanistic work with these cytoplasmic complexes will be necessary to more fully understand how this occurs and to identify relevance of these studies to metastatic disease.

Metastasis is clearly a relevant therapeutic target for patients with cancer. BRMS1 and SIN3 complexes regulate specific coding and non-coding metastasis associated genes depending on the specific composition of the complex. It follows that SIN3A:BRMS1 complexes are likely regulated by the environments surrounding cancer cells. This study has added an additional level of regulation for such complexes: subcellular localization. Although we have not identified mechanisms of trafficking between the nucleus and cytoplasm, we have identified important requirements for the metastasis suppressive function of BRMS1. These findings may help explain clinical studies of BRMS1 to further our understanding of the most deadly aspect of cancer.
